# Manipulating Microrobots Using Balanced Magnetic and Buoyancy Forces

**DOI:** 10.3390/mi9020050

**Published:** 2018-01-29

**Authors:** Lin Feng, Xiaocong Wu, Yonggang Jiang, Deyuan Zhang, Fumihito Arai

**Affiliations:** 1 School of Mechanical Engineering & Automation, Beihang University, Beijing 100191, China; linfeng@buaa.edu.cn (L.F.); xiaocongwu@buaa.edu.cn (X.W.); zhangdy@buaa.edu.cn (D.Z.); 2 Beijing Advanced Innovation Center for Biomedical Engineering, Beihang University, Beijing 100083, China; 3 Department of Micro-Nano Systems Engineering, Graduate School of Engineering, Nagoya University, Nagoya 464-0814, Japan; arai@mech.nagoya-u.ac.jp

**Keywords:** micro-robot, three-dimension manipulation, microfluidic chip

## Abstract

We present a novel method for the three-dimensional (3D) control of microrobots within a microfluidic chip. The microrobot body contains a hollow space, producing buoyancy that allows it to float in a microfluidic environment. The robot moves in the z direction by balancing magnetic and buoyancy forces. In coordination with the motion of stages in the xy plane, we achieved 3D microrobot control. A microgripper designed to grasp micron-scale objects was attached to the front of the robot, allowing it to hold and deliver micro-objects in three dimensions. The microrobot had four degrees of freedom and generated micronewton-order forces. We demonstrate the microrobot’s utility in an experiment in which it grips a 200 μm particle and delivers it in a 3D space.

## 1. Introduction

New technology in biomedical engineering, especially the manipulation of bioparticles (cells, biological tissue, etc.) in three-dimensional (3D) space, has attracted much attention [1]. A number of methods of manipulating bioparticles have been presented in the literature. Noncontact forces have been applied to bioparticle manipulations because they show great merit in terms of flexible actuation. Optical tweezers that use a highly focused laser beam to manipulate bioparticles are optional [2,3,4,5,6]. However, the output force of optical tweezers is not strong enough, especially for manipulating large-sized bioparticles. Dielectrophoresis (DEP), which uses a non-uniform electric field to exert forces on a dielectric particle [7], has been used for manipulating bioparticles [8,9,10]. However, Gray et al. reported that the electric fields used to rotate cells can cause cell damage [11]. In order to manipulate bioparticles, different kinds of microgrippers have been designed and actuated basing on static electricity [12], electro-thermal expansion [13], mechanical actuation [14,15], and other similar techniques [16,17,18,19]. Beyeler et al. [12] built an electrostatic microelectromechanical gripper containing a force sensor that can grip and release microspheres and HeLa cells in a two-dimensional (2D) plane. Colinjivadi et al. [13] presented a polymer “chopstick” gripper consisting of a metal heater layer and demonstrated nanoscale precision along the x, y, and z axes during cell manipulation. Ger et al. [20] presented a cell gripper based on magnetic zig-zag structures, which was actuated by a magnetic field. Wester et al. [15] designed a mechanically actuated microtweezer that allowed for movement in multiple degrees of freedom (DoF). Finally, Chung et al. [21] created a magnetically actuated microrobot capable of manipulating microgels in 3D space.

Bioparticle manipulation often leads to contamination because it is carried out in an open environment. Manipulations are more ideally performed in a closed space, such as inside a microfluidic chip [22]; unfortunately, the previously discussed micromanipulation systems were unable to manipulate particles within such spaces because the systems were too large. For example, the system presented by Chung et al., actuated by magnetic force, was large and complex because the actuator consisted of eight magnetic coils. Moreover, the system’s output force was too small to effectively manipulate larger objects, such as oocytes.

To make microrobots more practical and versatile, their size must be reduced for use in a microchip, and output forces must be increased. Applying a noncontact drive is a good method for solving these problems, as it significantly decreases robot size, making them more flexible for microchip use. Magnetic force is one of the most promising non-contact actuation methods for increasing output force and is minimally invasive (with respect to bioparticles). Microrobotic magnetic fields can be generated using a permanent magnet or electromagnetic coils. Electromagnetic coil systems occupy a large space and are more complicated, whereas magnetic fields generated by a permanent magnet are 10–100 times stronger with the same device size [23,24].

A few microrobots are currently capable of working in a 3D space, but their functionality is limited by their size and output force. We previously proposed a microrobotic system that can manipulate cells within a microchip, performing tasks such as the enucleation of bovine oocytes [25], accurate dispensing of single oocytes [26], 3D rotation of a single oocyte [27], and high-accuracy positioning [28]. In addition, we performed work in the area of micro-manipulation in a microchip [29].

**Figure 1:**
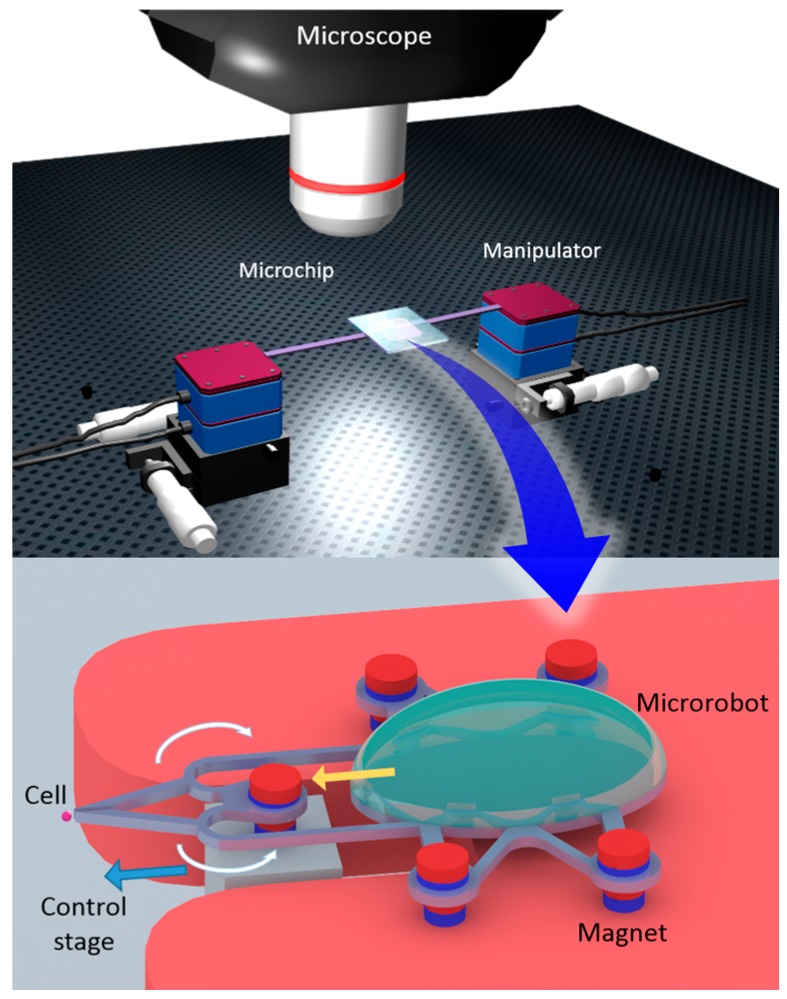
Conceptual overview of the microrobotic system. The robot is actuated by permanent magnets, and works in the aquatic environment of a microfluidic chip.

In this paper, we propose an innovative method for manipulating microrobots that are actuated by permanent fixed magnets on the manipulator, using magnetic and buoyancy forces. Figure 1 shows a conceptual overview of the microrobot system, where the robot contains a cavity for employing buoyancy in an aquatic environment. A controlled balance between buoyancy and magnetic forces allows for motion in the z direction, which (when combined with the motion of the xy plane) achieves 3D control. The noncontact drive allows the robot to be small enough for use in a microchip, and, using a gripper designed to sit in front of the robot, bioparticles can be easily manipulated. The microgripper is controlled by a stage corresponding to the gripper, a system that introduces opportunities for applications such as delivering objects to a target position or enucleating cells. Manipulation is performed under a microscope, preventing contamination within the enclosed environment. We demonstrate the delivery of a particle with a diameter of approximately 200 μm using this system. We also measured the distance between the two thin beams of the microgripper in various positions to show that it can grip particles of assorted sizes.

## 2. Materials and Methods

### 2.1. Microrobot Design

**Figure 2:**
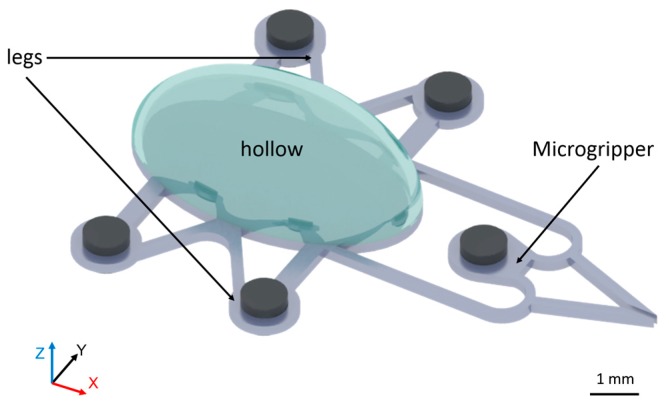
The design of the microrobot. A cavity is placed inside the main body of the robot to provide the buoyancy needed for control.

**Figure 3:**
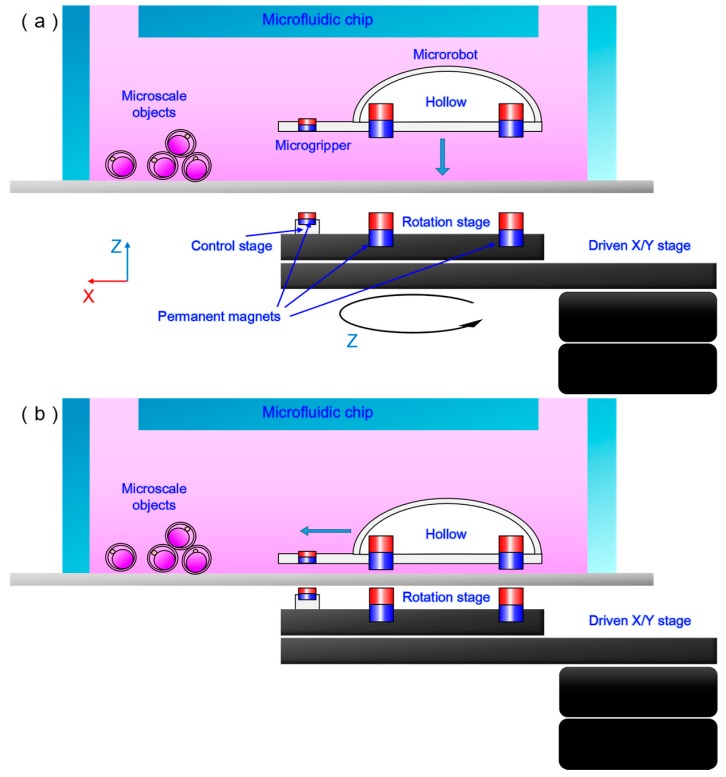

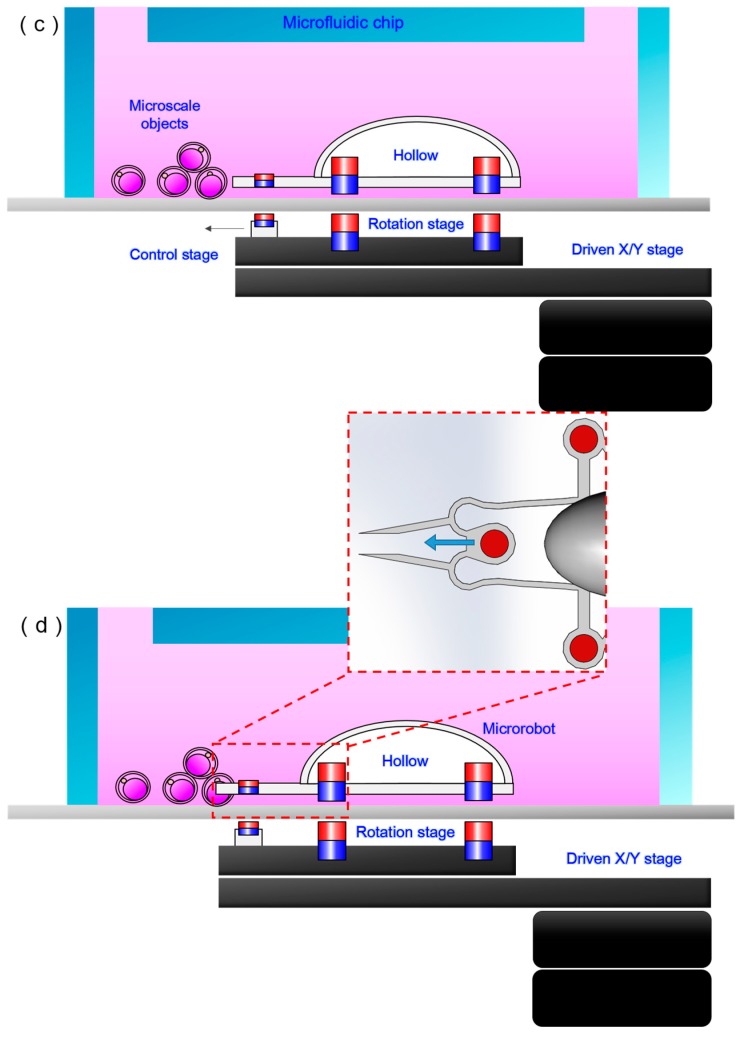
Schematic of the 3D microrobot control. The robot moves towards the stage when the stage moves towards the microfluidic chip, and when the stage moves away from the microfluidic chip, the robot moves away from the stage. (**a**,**b**) Control the robot to the intended position using the driven stage. (**c**,**d**) Move the control stage to adjust the state of gripper, to grip the microscale objects.

In consideration of the aquatic environment of cultured bioparticles, we present a method for 3D actuation in fluid using balanced magnetic and buoyancy forces. Figure 2 shows a microrobot with a cavity in its body for employing appropriate buoyancy force in an aquatic environment. As shown in Figure 2, four magnets permanently fixed to the microrobot’s legs play a key role in its 3D control. The magnet fixed to the microgripper controls the gripper. A schematic for the 3D control of the robot is shown in Figure 3.

Figure 3a–d show the process of gripping microscale objects by controlling the microrobot in 3D space. A driven stage is placed under the microrobot to control its 3D motion. The control stage, with a magnet fixed on it, can only move along the x axis on the driven stage. On the tip of the microrobot, a small permanent magnet is assembled in. Under the microfluidic chip, another permanent magnet according to where the magnet position on the tip of the micro-robot is put on the control stage. By controlling the magnet’s position on the stage, it is possible to control the opening and closing of the gripper.

When the driven stage moves towards the microchip, the distance between the stage and robot decreases, which increases the magnetic force and moves the robot toward the stage, as shown in Figure 3a. On the contrary, when the stage moves away from the microchip, the robot moves away from the stage. Thus, motion along the z axis can be achieved.

The relationship between buoyancy, magnetic force, and weight is a design consideration, so the microrobot’s total weight can be calculated by the formulas:

(1) $$G=G_{1}+G_{2}$$

(2) $$G_{1}=\rho_{1}gv_{1}$$

where *G*_1_ is the weight of the microrobot, *G*_2_ is the weight of the magnets fixed onto the microrobot, *ρ*_1_ is the density of photosensitive resin from which the microrobot is made, and *v*_1_ is the volume of the photosensitive resin.

The microrobot’s buoyancy can be calculated by the following formula:

(3) F=ρgv

where F is the microrobot’s buoyancy, *ρ* is the density of the liquid (water in this experiment), and *v* is the microrobot’s total volume.

The magnetic force between two cylindrical magnets can be approximated by the formula [30]:

(4) $$F\left(x\right)=\frac{\pi \mu_{0}}{4}M^{2}R^{4}\left[\frac{1}{x^{2}}+\frac{1}{{\left(x+2t\right)}^{2}}-\frac{2}{{\left(x+t\right)}^{2}}\right]$$

where *R* is the radius of the cylindrical magnets, *t* is their height, *M* is their magnetization, and *x* is the gap between them.

We experimentally determined the microrobot’s weight to be 4.738 × 10^−4^ N, and its buoyancy to be 6.325 × 10^−4^ N. As such, without a magnetic force, the robot floats in water. The maximum and minimum values of the magnetic forces are 0.0760 N and 8.59 × 10^−3^ N, respectively, when the robot is on the top and at the bottom of the liquid. When the robot is on top of the liquid, the magnetic force reaches its minimum, but is still sufficiently large to control the robot’s movement, confirming the theoretical feasibility of the actuator method.

### 2.2. Microgripper Simulation

By moving the control stage, the distance between the magnets in the control stage and the micro-gripper changes, altering the strength of the magnetic field at the microgripper’s location. Therefore, the gap state and separation distance of the gripper can be controlled. In our experiments, the component force along the x axis was hard to measure during experiments, as shown in Figure 3, and was experimentally evaluated using the load cell (LVS-5GA, Kyowa Electronic Instruments Co., Ltd. Tokyo, Japan). The maximum component force along the x axis was approximately 0.03 N. We conducted a gripper control simulation, wherein the component force applied to the microgripper was 0.03 N.

**Figure 4:**
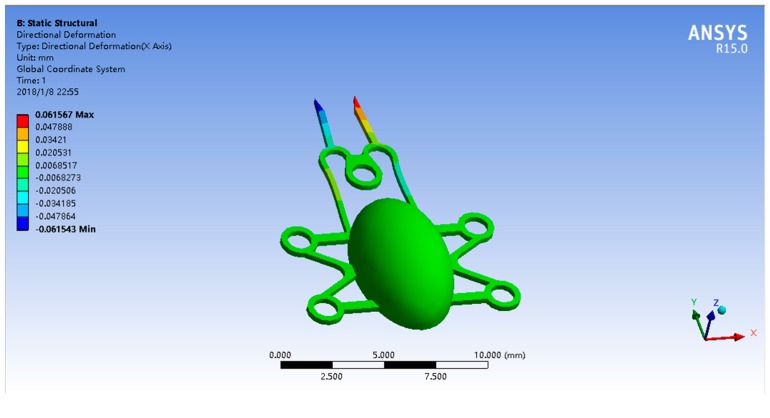
Gripper control simulation. The gap between the two thin beams increases approximately 123 μm compared to the original position; compared to the size of bioparticles, this value is sufficient for manipulation.

Figure 4 shows the simulation wherein a magnetic force of 0.03 N was applied to the microgripper in the +y direction. The gap increased by approximately 123 μm, for a total of 273 μm, which is sufficient for manipulating bioparticles.

### 2.3. Experimental Setup

**Figure 5:**
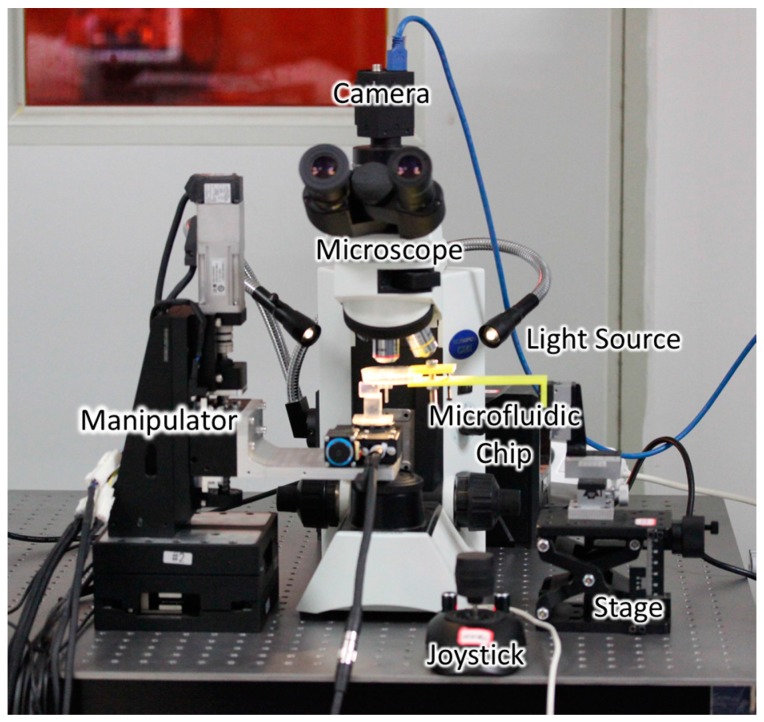
Overview of the manipulation system.

Figure 5 shows an overview of the manipulation system, consisting of the observation system and the control system. To observe the manipulation better, the experiment was performed under a camera-attached microscope with an external light source added. The microfluidic chip was fixed on a stage, and the manipulator was operated with a joystick. The equipment mentioned above was fixed to a shock-proof platform to meet the need for high positioning accuracy.

The robot was fabricated via a high-quality 3D printing process (Form2, Formlabs Co., Ltd., MA, USA) using a photosensitive resin (Clear V2, Formlabs Co., Ltd., MA, USA). The microrobot can be 3D-printed directly. However, the hollow inside the robot was full of liquid photosensitive resin due to the liquid manufacturing environment. To empty the hollow chamber, a hole of around 0.6 mm in diameter was left open through which the liquid photosensitive resin could be removed and it was sealed later. The driven and control stages (to which the magnets were fixed) were manufactured by the same 3D printing process. The magnets were fixed to the microrobot, and the stages using a liquid photosensitive resin were fixed by exposure to UV light.

A four-DoF stage (HEIDSTAR Co., Ltd. Fujian, China) was used in the experiment. Its precision is 300 nm, providing enough accuracy compared to the size of the bioparticles and microrobot.

A microfluidic chip consisting of dimethyl siloxane (PDMS, DOW CORNING Co., Ltd. Wiesbaden, Germany) and a glass substrate served as the experiment platform. The cover of the chip was fabricated from PDMS, and the microfluidic chip was filled with water. The robot and bioparticles were encapsulated within the microfluidic chip to protect the particles from contamination. The channel of the microfluidic chip was designed specifically for the requirements of this experiment.

The experiment was observed using a microscope (CX41, OLYMPUS Co., Ltd., Tokyo, Japan) with a mounted camera (GS3-U3-23S6C-C, POINTGREY Co., Ltd., BC, Canada) set above the microfluidic chip.

## 3. Results

**Figure 6:**
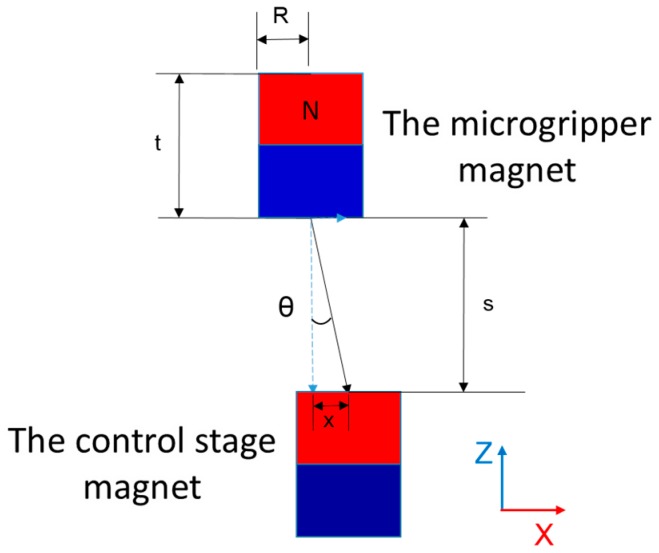
Actuation of the microgripper, triggered by the control stage magnet sweeping along the x axis.

Figure 6 shows the actuation of the microgripper as triggered by the control stage magnet (correspondingly sweeping along the x axis). By moving the control stage magnet along the x axis, its position relative to the micro-gripper magnet changed. A component force along the x axis was produced during this progress. This component force controlled the microgripper’s opening and closing. The extent to which the control stage magnet moved determined the magnitude of the component force along the x axis, which in turn changed the distance between the two thin beams of the microgripper, allowing it to grip and release different-sized bioparticles.

**Figure 7:**
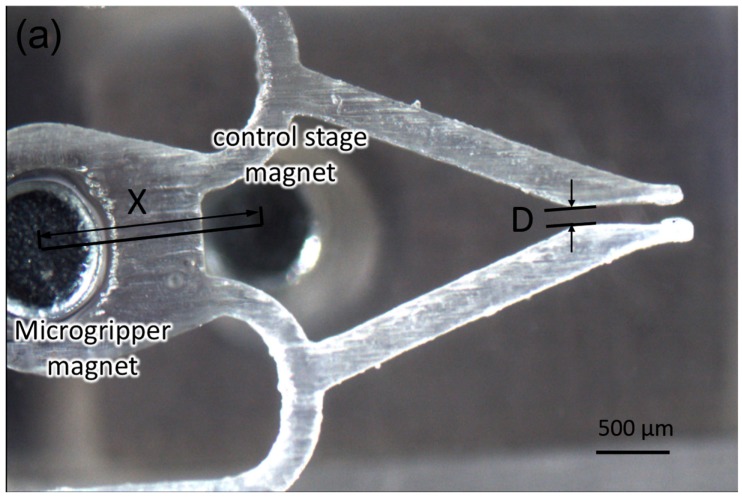

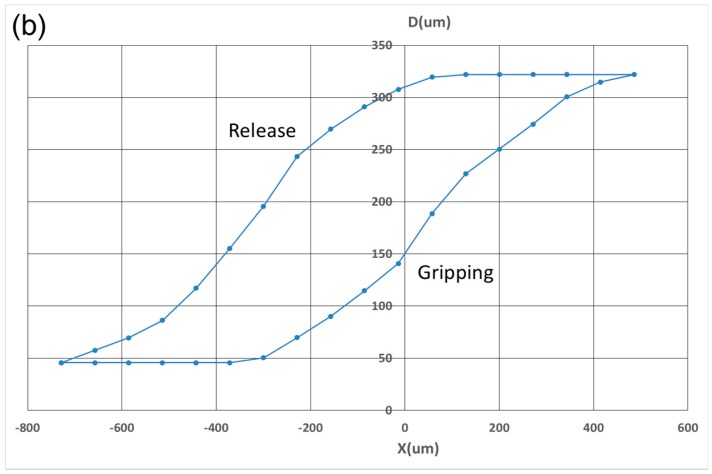
Relationship between magnet movement and the gap between the two thin beams of the microgripper. (**a**) An illustration of the experiment. The distance between the two magnets fixed to the microgripper and control stage is x; D is the gap in the gripper. (**b**) The relationship between the moving of the magnet x and the size of the gripper opening D.

The relationship between the change in the magnets’ distance and the gap between the microgripper was examined experimentally, as shown in Figure 7a. The distance between the control stage and microgripper magnet was measured from the center of each magnet on the x axis (as shown) and gripper gap distance D was measured. Figure 7b shows that the rate of increase of D depends on the magnet position (specifically, the value of x). When a microrobot is driven by a permanent magnet beneath the glass substrate, there is a distance in which the microrobot does not follow the drive magnet; we named this distance the “dead band” [31] and it can be seen in the graph. The dead band is caused by the static friction of the microgripper with the glass substrate. This shows that there is a hysteresis caused by the dead band between the gripping phase and release phase in Figure 7b.

As shown in Figure 6, the force along the x axis actuated the microgripper. When angle θ was zero, there was no component force along the x axis, leaving it in its original position. When the control stage magnet moved along the x axis, θ increased and a component force was applied to the gripper along the x axis. The two thin beams on both sides of microgripper were subsequently bent out of shape, causing the gripper to open or close. The size of the opening corresponded to the magnitude of the component force. When the displacement of the control stage magnet was small, the magnetic force decreased only slightly, but θ increased quickly, as shown in Figure 7b. When the displacement was close to zero, the slope of the curve was large, meaning that the gripper’s gap increased quickly. With increasing displacement, the increase in the y axis slowed, owing to the geometrically related increase in θ. When displacement exceeded a certain range, the magnetic force decreased rapidly, even though θ continued to increase; this became the most important influence, and the curve’s slope decreased. At a specific displacement, the increase in θ and the decrease in magnetic force were balanced—where the slope of the curve was zero and the value of the y axis (gap distance D) was maximized. As shown in Figure 7b, D ranged from 50 to 320 μm, meaning that the microgripper was suitable for manipulating large bioparticles. According to the graph, we can select size x to obtain the corresponding D when manipulating different-sized bioparticles.

**Figure 8:**
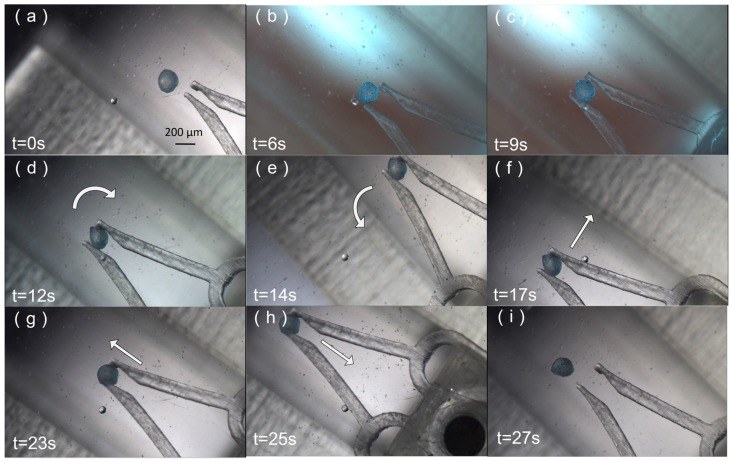
The microgripper grasping and carrying a microparticle. (**a**) The microgripper is in its original state. (**b**) The microgripper ready to be opened. (**c**) The gripper open under action of a magnetic force applied to the gripper. (**d**) The robot is rotated clockwise, carrying the particle. (**e**) The microgripper rotates counter-clockwise. (**f**–**i**) The microgripper carries the micro-particle along a straight line and release the microparticle.

Figure 8 shows the result of the experiment wherein the micro-gripper grasped and carried a particle. Figure 8a is an image of the micro-gripper in front of the robot, in its original state. When the corresponding control stage magnet moved in the +x direction, a component force in +x was applied to the microgripper. The applied force stretched the thin beams on both sides of the microgripper out of shape, opening the gripper in preparation to grab the particle. When the opening gap of the tip was large enough to grab the particle, the gripper moved right, and then could close the tip to grab the particle (i.e., the control stage magnet moved in the –x direction).

To transport a particle and perform other manipulations, the microrobot must be controlled in 3D space. Motion in the z direction was achieved by balancing the magnetic and buoyancy forces. Motion in the xy plane and rotation around the z axis depended on the motion of the 4-DoF stage. Four other magnets permanently fixed to the microrobot play an important role in 3D control. After moving the robot to the desired position, a reversal of the gripper process opens the gripper and releases the bioparticle.

**Figure 9:**
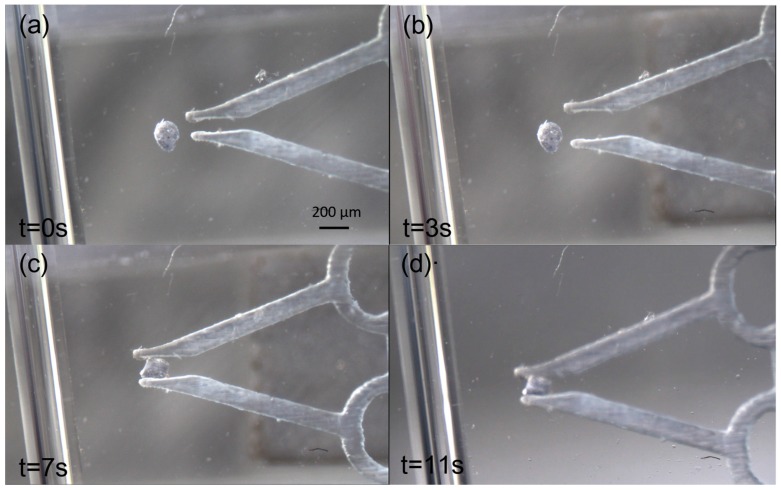
Sequential micrographs of the microgripper manipulation experiment. (**a**) The microgripper is in position to open and grip. (**b**) The microgripper opens under the action of a magnetic component force applied to the gripper. (**c**) The microgripper grips the particle by decreasing the component force. (**d**) The microrobot changes height (it appears dimmer in the image because it has risen in the fluid as a result of changes to the balanced magnetic and buoyancy forces).

Figure 9 shows sequential micrographs of the microgripper manipulation experiment. A circular tube with a diameter of 840 μm is located on the left side of the graph in Figure 9, indicating the changing height of the robot in the microfluidic chip. At first, the particle and robot were in their original positions, as shown in Figure 9a. The gripper in front of the microrobot opened to grip the particle by controlling the control stage magnet, as shown in Figure 9b. In Figure 9c, the particle is being gripped. In Figure 9d, the robot and particle changes heights using a balance between magnetic and buoyancy forces. The robot was out of the focal plane; the microrobot tip becomes dimmer in Figure 9d compared to other images. Hence, the microrobot floated and its height in the liquid was controlled by the magnetic force.

## 4. Discussion

In the first experiment, we examined the relationship between magnet movement and the gap between the two thin beams of the microgripper. The results of this work demonstrate 3D microparticle gripping and transport by innovative actuated microrobots. Figure 7 illustrates this relationship, showing the microrobot’s strong adaptability in manipulating a large range of microparticle sizes—from 50 to 320 μm. Furthermore, the demonstration of microparticle grasping and transportation suggests that the microrobot could be successfully controlled by balancing buoyancy and magnetic forces.

## 5. Conclusions

In this paper, we have described an innovative microrobot manipulation method using balanced magnetic and buoyancy forces in a microfluidic chip. Compared to existing methods, it suffers from less contamination because it functions in a closed space, owing to its small size and noncontact actuation principle. Because of its strong output force, and wide gripping range, it offers the ability to manipulate objects of varying sizes. In addition, the liquid manipulation environment was extremely suitable for bioengineering. In this experiment, we successfully demonstrated that this method is a promising tool for single-cell manipulation. Our future work will strengthen the motion stability on the z axis and add more automation to the microrobot system.

## Supplementary Materials

The following are available online at http://www.mdpi.com/2072-666X/9/2/50/s1, Video S1: Microparticle manipulations.
